# The New Mesmerism

**Published:** 1893-05

**Authors:** 


ARTICLE VII.

### THE NEW MESMERISM.

A Paris correspondent of the London *Times*, who has been studying hypnotism as practiced by Dr. Charcot and his followers during the last decade, details some curious achievements of the hypnotic art. He insists, however, in the outset, that hypnotism of to-day, under all its aliases, is identical with the "mesmerism" or "animal magnetism" of former times. The marvels of the present are merely, he affirms, a fresh revival of the practices originally started in a systematic way by Mesmer over a hundred years ago and subsequently brought into notice with improvements in 1820, and again about 1845. It was in 1845 that Braid, the Manchester surgeon, having learned the art of Mesmer from a professional mesmerist, gave it the name hypnotism. But nothing has been changed save the name. The hypnotism of today is the mesmerism of the last century, only with each successive revival of interest in it its strange manifestatious are studied with the help of a fuller knowledge of physiology and pathology. None of the new feats of hypnotists are new in principle. The extraction of teeth under hypnotic anæsthesia and the treatment of dipsomania by "suggestion" are repetitions of experiments fifty years old. All that is new, perhaps, is the effort now being made to utilize hypnotism as a system of medical treatment.

The present method of producing the hypnotic state is to seat the patient in a comfortable arm-chair, with his back to the light, and direct him to gaze at a revolving mirror placed on a stand immediately in front of him, one foot from his face, and rather above the level of his eyes. The mirror, of bright tin, about eight inches long, is revolved by means of a common clock-work arrangement in a box below, the revolutions being about 200 a minute. With each revolution the light flashes across the patient's eyes. The effect varies with the susceptibility of the patient. Some fall at once into an unconscious condition, with eyes wide open, staring fixedly at the machine, the face expressing some vivid emotion. In other cases, after five, ten or twenty minutes, the hypnotist gently closes the patient's eyes and says, "Go to sleep." Some obey and some do not. Unless one is uncommonly self-willed he is likely to be affected by the light flashing into his eyes several times a second. The repeated stimulus of the optic nerve causes a distraction of mind, which renders it impossible to concentrate attention upon anything. The thinking faculty will not work. It lies inert and half-bewildered; control is lost. This being its condition when the hypnotist closes the eyelids and says firmly, "Go to sleep," the idea is seized with avidity as a means of relief, and is held tenaciously till unconsciousness is reached.

The mirror is not indispensable. It is enough to gaze steadily for awhile at any bright object—a piece of metal or the hypnotist's eyes. Those mentally feeble folk who are open to every external influence, and find it easier to follow another's idea than to originate one of their own, are usually hypnotized without difficulty. Some ultra-hysterical organizations pass spontaneously into the hypnotic state, but these are rare. With those who have been frequently hypnotized only some very simple proceeding is necessary. It is enough for the operator to place his finger on the patient's forehead and say: "Look at me!" and afterward quietly close his eyes. Just what the hypnotic state is no one can yet tell. It evidently implies, however, a disordered train. Theorists in Paris speak of distinct varieties of this disorder as "lethargy," "catalepsy," "somnambulism;" &c. Others say only "light sleep," "sleep, sleep," very deep sleep," &c. Really these distinctions signify nothing, as the phenomena do not occur in a definite and orderly manner. But two hypnotic conditions are generally recognized, namely, sleep and somnambulism. It is the latter which supplies the marvels of heightened faculties and realized suggestions. The brain of the somnambulist becomes capable of a thousand things wholly impossible in its natural state, It is asserted by the *Times'* correspondent to be beyond question that a plain woman, ordinarily dull and ignorant, will, when hypnotized, realize admirably a suggested character, and play the part with an utter truthfulness that surpasses the art of a most accomplished actress.

A new phenomena, apparently, is the transference of sensibility from a hypnotic subject to inanimate objects. The correspondent tells of a woman, who, when hypnotized, felt pinches given to a gutta percha statuette, and felt them in such parts of her body as corresponded with those in which the gutta percha figure was pinched. This hysterical young woman was hypnotized in the usual way. The figure, about a foot long, was at first held in front of her. Then it was placed out of her sight and pinched. Sometimes she felt the pinches and sometimes she did not. All this time the hypnotist had his hand in contact with her. When he held the figure where she could see it she obviously felt the pinches given it very acutely. When the sole of the figure's foot was touched she was tickled beyond endurance. In one experiment the figure was placed on a table out of sight both of the girl and the operator, while another gentleman put one hand on the operator's back and the other on the image. Whenever the second gentleman touched the figure the girl felt it. In another experiment she felt the pinching when the operator was some feet distant from her and the figure was out of sight. She always felt the pinches, but not always where they were inflicted on the mannikin. Such is one of the latest wonders of Parisian hypnotism.—*Baltimore Sun.*

